# Supplementary material for: Acyl-lipid desaturases and Vipp1 cooperate in cyanobacteria to produce novel omega-3 PUFA-containing glycolipids
Source: Biotechnol Biofuels. 2020 May 6;13:83. doi: 10.1186/s13068-020-01719-7 (PMC7203895; doi:10.1186/s13068-020-01719-7)
Supplement: Supplementary file 1 — Additional file 1. Supplemental information reporting bioinformatics, as well as additional fatty acid profile and lipidomics data for all three cyanobacterial strains. [file 13068_2020_1719_MOESM1_ESM.pdf]

Acyl-lipid desaturases and Vipp1 cooperate in cyanobacteria to produce novel omega-3 PUFA-containing glycolipids

Leslie B. Poole[1,2,*], Derek Parsonage[1,2], Susan Sergeant[1,], Leslie R. Miller[3,7], Jingyun Lee[4,5], Cristina M. Furdui[2,4,5], Floyd H. Chilton[3,5,6,*]

[1]Department of Biochemistry, [2]Center for Redox Biology and Medicine, [3]Department of Physiology and Pharmacology, [4]Department of Internal Medicine, Section on Molecular Medicine, and [5]Comprehensive Cancer Center, Wake Forest School of Medicine, Winston-Salem, NC 27157, and [6]Department of Nutritional Sciences and the BIO5 Institute, University of Arizona, Tucson, AZ; [7]present address 139 N St. Patrick St., New Orleans LA 70119

Supplementary Material

Included below are additional results related to Bioinformatics, total fatty acid contents in all three strains of cyanobacteria studied (Fig. S1), contributions of individual genes to the PUFA-promoting effect of pDBV (Fig. S2), lipidomics data from the second experiment (Table S1) and the identity of the second acyl chain in n-3 PUFA-containing lipids (Fig. S3), summary of ALA and SDA production by pBV and pDBV-containing *Leptolyngbya* sp. strain BL0902 (Table S2), and sample MS2 spectra used for peak identification (Fig. S4). Two additional Excel spreadsheets of supplementary data are also included:

Additional file 2. Fatty acid profile for cyanobacteria with and without plasmid constructs (Excel)

Additional file 3. Identification of glycoglycerolipids in lipid extracts of wild type and genetically-engineered cyanobacteria (Excel)

Results

Bioinformatics to search for *des*B and *des*D loci in *Leptolyngbya* species. To complement our fatty acid analyses of wild type *Leptolyngbya* sp. strain BL0902 for which there is not yet a published sequence, we conducted searches for the well conserved Δ6 (*des*D) and ω3 (*des*B) desaturase genes based on the known sequences from *Synechocystis* sp. PCC6803, as well as variants from several

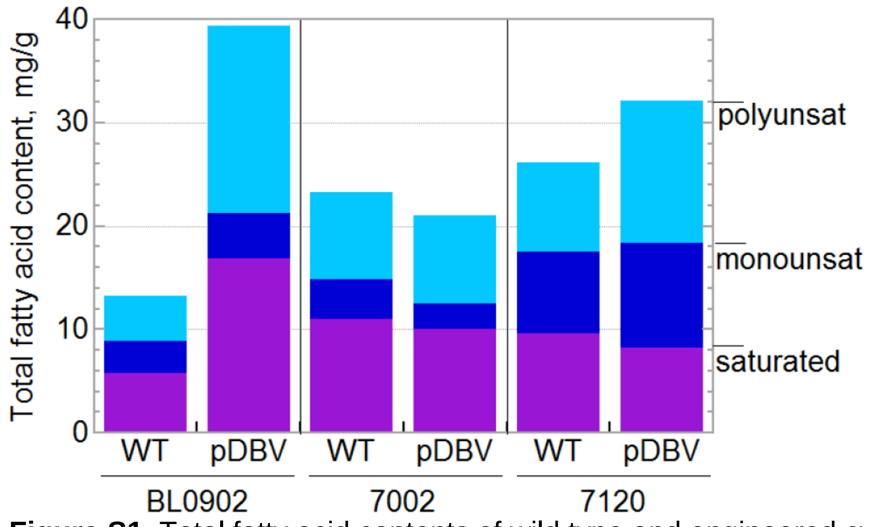

Figure S1. Total fatty acid contents of wild type and engineered cyanobacterial strains. Three cyanobacterial strains, *Leptolyngbya* sp. strain BL0902, *Synechococcus* sp. PCC 7002 and *Anabaena* sp. PCC 7120, were selected for testing given their favorable starting contents of linoleic acid (18:2). Wild type (WT) or engineered (+pDBV) strains were grown, harvested and analyzed for fatty acid content (FAME analysis) as described in Fig. 1 and Methods. pDBV includes three cyanobacterial genes encoding DesD (Δ6 desaturase), DesB (omega-3 or Δ15 desaturase), and the "vesicle-inducing protein in plastids" (Vipp1). Shown are total saturated (magenta), monounsaturated (dark blue) and polyunsaturated (cyan) fatty acid contents (n=5, 3, 3, 3, 2 and 1 from left to right).

other cyanobacterial species to confirm the results. A Blastp search for *des*B in *Leptolyngbya* species at the National Center for Biomedical Information (https://blast.ncbi.nlm.nih.gov/) in March 2020 returned 22 very convincing homologues (e-value < 2E-168) using the non-redundant protein sequence database (nr), whereas no convincing *des*D ortholog was identified in a parallel search (e-values all > 1E-14).

Supplemental data reporting cyanobacterial fatty acids and lipids. Reported below are total fatty acid contents in all three strains of cyanobacteria studied (Fig. S1), contributions of individual genes to the PUFA-promoting effect of pDBV (Fig. S2), and the identity of the second acyl chain in n-3 PUFA-containing lipids based on the lipidomics analysis (Fig. S3).

Lipidomics of *Leptolyngbya* sp. strain BL0902 and *Synechococcus* sp. PCC7002. Analyses were conducted as described in the main manuscript, with wild type and pDBV-containing forms of each of two cyanobacterial strains (n=1 each). In this second experiment, ALA, SDA and ETA were found in a larger number of lipid species, and SQDG species were more enriched in SDA and particularly ALA in this experiment, whereas PG rather than SQDG contained measureable amounts of these fatty acids in the first experiment (Table S1).

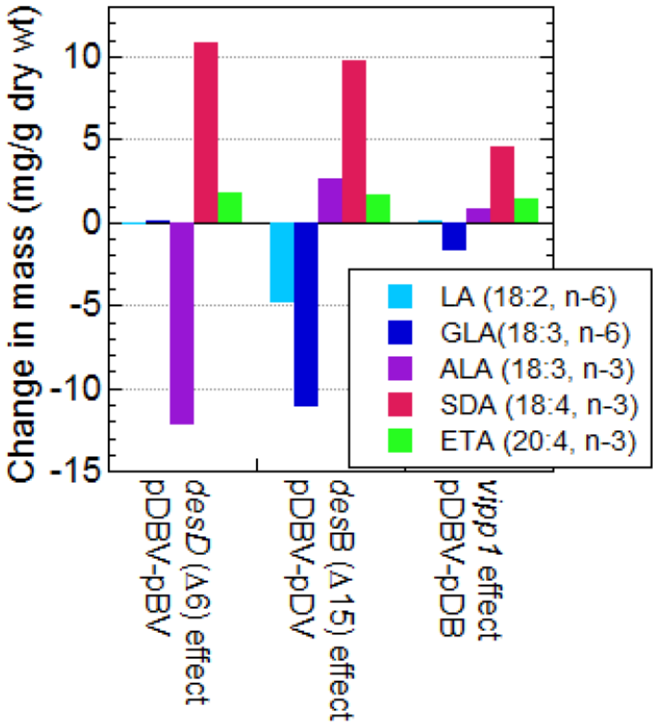

Figure S2. Individual contribution toward the PUFA profile (generated by pDBV addition) of the engineered cyanobacterial genes in the context of the presence of the other two genes. Based on FAME analysis of the PUFAs depicted in Figure 3, changes in mass of each of the 18C- and 20C-PUFAs are shown, with conversions observed consistent with each of the expected molecular functions.

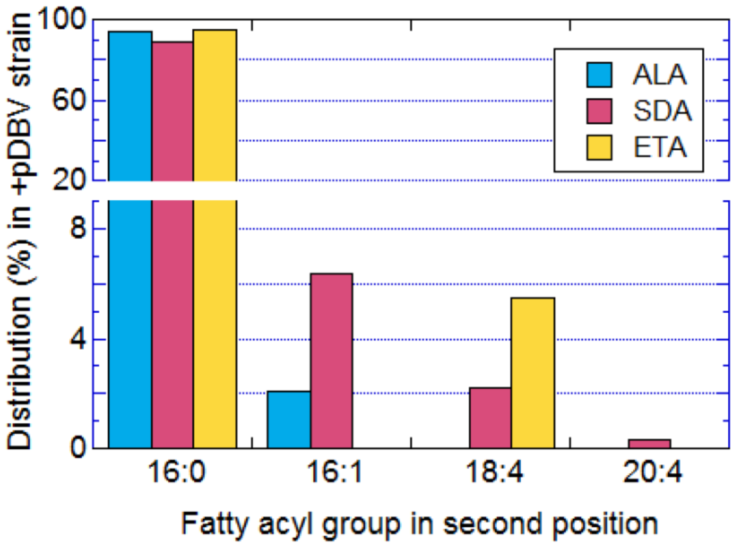

Figure S3. Selective distribution of major n-3 PUFAs (ALA, SDA and ETA) within *Leptolyngbya* BL0902 glycolipids with respect to the nature of the second of the two acyl chains within each lipid molecule, based on lipidomics analyses depicted in Figure 4.

Table S1. Molecular species of lipids by LC-MS/MS in wild type (WT) and engineered (+pDBV) *Leptolyngbya* sp. strain BL0902 and *Synechococcus* sp. PCC7002 (n=1 each).[a]

Molecular species	Cyano strain	MGDG		DGDG		SQDG		PG	
		WT	+pDBV	WT	+pDBV	WT	+pDBV	WT	+pDBV
14:0/16:0	BL0902	0.111	0.850	0.000	0.000				
	7002	0.225	0.112	0.012	0.008				
14:0/18:3	BL0902	0.008	0.042	0.012	0.008			0.000	0.009
	7002	0.017	0.008	0.015	0.001			0.001	0.010
14:0/18:4	BL0902	0.000	0.168	0.001	0.012				
	7002	0.000	0.022	0.003	0.004				
16:0/16:0	BL0902	0.051	0.796	0.000	0.012	0.005	0.006	0.001	0.256
	7002	0.053	0.030	0.000	0.004	0.027	0.008	0.015	0.032
16:0/16:1	BL0902	0.090	0.508	0.001	0.045			0.003	0.105
	7002	0.072	0.022	0.002	0.004			0.018	0.039
16:0/16:3	BL0902	0.000	0.051						
	7002	0.000	0.014						
16:0/17:2	BL0902	0.019	0.065					0.000	0.010
	7002	0.057	0.030					0.011	0.005
16:0/17:4	BL0902	0.000	0.024						
	7002	0.000	0.015						
16:0/18:1	BL0902	0.629	1.479	0.000	0.000			0.207	1.164
	7002	0.676	0.273	0.005	0.003			0.640	0.353
16:0/18:2	BL0902	0.566	3.214	0.011	0.134			0.057	1.843
	7002	3.964	0.979	0.030	0.056			1.710	0.266
16:0/18:3	BL0902	0.168	1.582	0.017	0.058	0.128	2.301		
	7002	0.692	0.858	0.325	0.011	0.796	4.914		
16:0/18:4	BL0902	0.001	5.330	0.012	0.212	0.004	1.109	0.000	0.035
	7002	0.001	2.199	0.126	0.123	0.000	0.235	0.000	0.013
16:0/18:5	BL0902	0.001	0.009						
	7002	0.025	0.008						
16:0/19:2	BL0902	0.007	0.011						
	7002	0.029	0.005						
16:0/20:4	BL0902	0.053	0.527	0.000	0.712			0.000	0.114
	7002	0.015	0.145	0.000	0.295			0.000	0.035
16:0/20:5	BL0902			0.006	0.038	0.031	0.023		
	7002			0.003	0.011	0.019	0.023		
16:1/18:2	BL0902	0.483	0.122	0.000	0.000			0.010	2.403
	7002	0.580	0.021	0.003	0.000			0.464	2.173

16:1/18:3	BL0902	0.100	0.180	0.048	0.014	0.014	0.088	0.017	0.046
	7002	0.161	0.163	0.004	0.004	0.001	0.059	0.018	0.057
16:1/18:4	BL0902	0.001	1.006	0.013	0.467				
	7002	0.000	0.363	0.116	0.282				
16:2/18:3	BL0902			0.000	0.025				
	7002			0.000	0.010				
16:2/18:4	BL0902	0.013	0.117	0.000	0.014				
	7002	0.003	0.053	0.005	0.008				
16:3/18:4	BL0902	0.000	0.021	0.000	0.053				
	7002	0.000	0.011	0.000	0.033				
17:1/18:2	BL0902	0.019	0.003						
	7002	0.051	0.003						
17:1/18:4	BL0902	0.000	0.016						
	7002	0.000	0.017						
17:2/18:4	BL0902	0.000	0.014	0.000	0.000				
	7002	0.000	0.011	0.003	0.002				
17:3/18:4	BL0902	0.000	0.007	0.000	0.003				
	7002	0.000	0.008	0.000	0.004				
18:0/18:4	BL0902	0.005	0.012						
	7002	0.001	0.011						
18:1/18:1	BL0902	0.199	0.030	0.000	0.000	0.090	0.028	0.018	0.306
	7002	0.081	0.013	0.004	0.001	0.134	0.030	0.070	0.014
18:1/18:2	BL0902	0.479	0.114	0.007	0.000			0.015	0.050
	7002	0.272	0.070	0.005	0.000			0.041	0.005
18:1/18:4	BL0902	0.194	0.470						
	7002	0.222	0.256						
18:1/18:5	BL0902					0.003	0.065		
	7002					0.002	0.065		
18:1/20:4	BL0902	0.000	0.014						
	7002	0.002	0.007						
18:2/18:2	BL0902	0.330	0.027	0.029	0.000			0.003	0.017
	7002	0.554	0.041	0.032	0.001			0.008	0.017
18:2/18:3	BL0902	0.079	0.207	0.028	0.019				
	7002	0.281	0.106	0.186	0.013				
18:2/18:4	BL0902	0.005	0.097	0.011	0.026				
	7002	0.001	0.022	0.127	0.031				
18:2/18:5	BL0902					0.000	0.088		
	7002					0.000	0.031		

18:2/20:4	BL0902	0.000	0.019						
	7002	0.001	0.015						
18:3/18:3	BL0902	0.013	0.405	0.000	0.020			0.000	0.001
	7002	0.030	0.094	0.000	0.014			0.001	0.026
18:3/18:4	BL0902	0.000	0.502	0.010	0.087				
	7002	0.001	0.261	0.015	0.063				
18:3/20:3	BL0902	0.000	0.006						
	7002	0.000	0.009						
18:3/20:4	BL0902	0.000	0.029	0.000	0.210				
	7002	0.000	0.016	0.000	0.111				
18:4/18:4	BL0902	0.000	0.868	0.000	0.014	0.000	0.075		
	7002	0.000	0.203	0.000	0.013	0.000	0.006		
18:4/20:4	BL0902	0.000	0.114	0.000	0.167				
	7002	0.000	0.048	0.000	0.049				
18:4/20:5	BL0902	0.003	0.020						
	7002	0.002	0.014						

[a]Values reported are estimates of mg/g of total fatty acid based on (i) normalized peak areas from LC/MS, (ii) fraction of total peak area for each species in a sample, and (iii) known total fatty acid yield for that organism from GC-FID analysis. This treatment assumes that all species exhibit the same ionization efficiency. Shown are mean +/- standard deviation for wild type (WT) *Leptolyngbya* BL0902 without and with pDBV, and *Synechococcus* sp. PCC 7002 (n=1 each). No entry means that the species was not observed in the WT or pDBV samples in that category.

[b]Molecular species shown are for the two fatty acyl chains, giving carbon chain length and number of double bonds for each.

[c]MGDG = monogalactosyldiacylglycerol; DGDG = digalactosyldiacylglycerol; SQDG = sulfoquinovosyldiacylglycerol; PG = phosphatidyl glycerol

Table S2. Content (reporting mass and mol% of total fatty acids) of 18-carbon omega3 fatty acids in engineered *Leptolyngbya* sp. strain BL0902.

Species		ALA (18:3, n-3), mg/g and mol% of total FAs[a]	SDA (18:4, n-3) mg/g and mol% of total FAs[a]
Leptolyngbya sp. strain BL0902	None (wt)	3.1 ± 1.6 mg/g<br>22.6 ± 11.5 %	n.d.[b]
	With pBV (to express DesB and Vipp1)[c]	16.0 ± 0.7 mg/g<br>37.7 ± 1.7 %	n.d.
	With pDBV (to express DesD, DesB and Vipp1)[c]	3.8 ± 0.4 mg/g<br>9.3 ± 0.9 %	10.8 ± 0.4 mg/g<br>26.6 ± 1.0%

[a] Expressed as mean ± standard deviation.

[b] n.d. = none detected

[c] pBV encodes *Synechococcus* sp. PCC7002 DesB for Δ15 desaturase (also known as ω3 or methyl end desaturase) and *Synechococcus* sp. PCC7002 Vipp1 for inducing thylakoid membranes; pDBV is the three-gene plasmid derived from pAM4418 (Fig. 2) developed in this work which includes the two genes of pBV plus *Synechocystis* sp. PCC6803 *des*D for Δ6 desaturase expression.

Accumulation of ALA in *Leptolyngbya* BL0902 with pBV. Although our goal in this study was to achieve significant SDA production by cyanobacteria, ALA is also a potentially important omega-3 fatty acid with greater stability that EPA and DHA. ALA accumulated in these studies only in the absence of *des*D (the Δ6 desaturase); our pBV plasmid enabled significant levels of up to 37.7% to accumulate. Similar experiments by others at relatively low illumination like ours [$\leq$50 µmol photons/(m^2 · s)] achieved about 23 to 28% ALA accumulation, an amount that is similar to that present in wild type *Leptolyngbya* BL0902 (Table 3). Yoshino et al. (1) reported accumulation of as much as 53% ALA under conditions of high incident light [150 µmol photons/(m^2 · s)], but in those experiments (which included *des*D overexpression), SDA reached a maximum of only 6.2%.

Confirmation of mass spectrometry peak identification through sample MS-MS spectra, and combined approaches to track SDA distribution among lipid species. As an illustration of the

quality of the MS-MS data used for identification of lipid species in the cyanobacterial samples, two MS2 spectra of key species of interest (but at relatively low abundance) are shown along with labeling of several of the major peaks that provide the information for identification (Fig. S4). An Excel file is also provided which contains information on delta mass units in ppm to indicate the accuracy of identification.

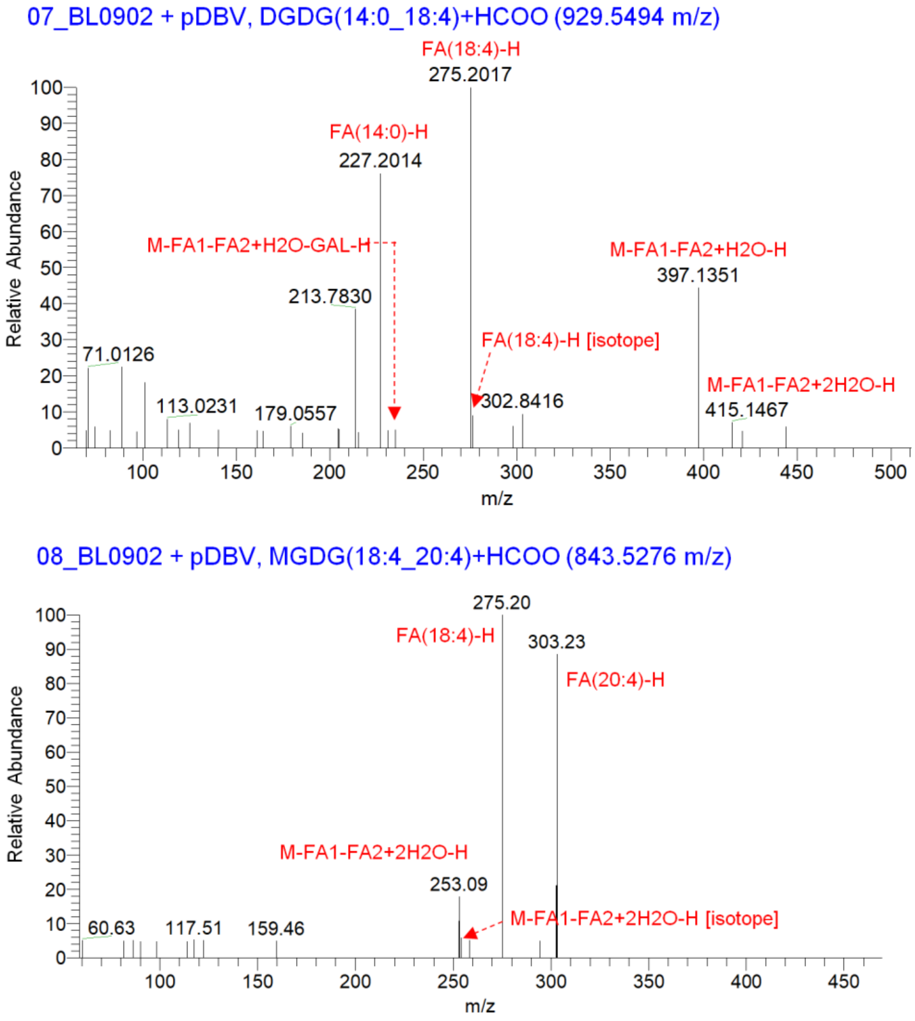

Figure S4. Spectra from MS-MS of two lipid species of interest. Shown are the fragment ions and identifying features for lipids from two samples of *Leptolyngbya* sp. strain BL0902 with pDBV. The upper panel shows DGDG with 14:0 and 18:4 fatty acyl chains (parent ion 929.5494 m/z), and the lower panel shows MGDG with 18:4 and 20:4 acyl chains (parent ion 843.5276 m/z). All fragments labeled in red are perfect matches.

Reference

1. Yoshino T, Kakunaka N, Liang Y, Ito Y, Maeda Y, Nomaguchi T, Matsunaga T, Tanaka T. Production of omega3 fatty acids in marine cyanobacterium *Synechococcus* sp. strain NKBG 15041c via genetic engineering. Appl Microbiol Biotechnol. 2017;101:6899-905.
